# Immune Activation Promotes Evolutionary Conservation of T-Cell Epitopes in HIV-1

**DOI:** 10.1371/journal.pbio.1001523

**Published:** 2013-04-02

**Authors:** Rafael Sanjuán, Miguel R. Nebot, Joan B. Peris, José Alcamí

**Affiliations:** 1 Institut Cavanilles de Biodiversitat i Biologia Evolutiva, Universitat de València, València, Spain; 2 Departament de Genètica, Universitat de València, València, Spain; 3 Instituto de Física Corpuscular, Universitat de València-Consejo Superior de Investigaciones Científicas (CSIC), València, Spain; 4 AIDS Immunopathogenesis Unit, Instituto de Salud Carlos III, Madrid, Spain; Imperial College London, United Kingdom

## Abstract

The immune system should constitute a strong selective pressure promoting viral genetic diversity and evolution. However, HIV shows lower sequence variability at T-cell epitopes than elsewhere in the genome, in contrast with other human RNA viruses. Here, we propose that epitope conservation is a consequence of the particular interactions established between HIV and the immune system. On one hand, epitope recognition triggers an anti-HIV response mediated by cytotoxic T-lymphocytes (CTLs), but on the other hand, activation of CD4^+^ helper T lymphocytes (T_H_ cells) promotes HIV replication. Mathematical modeling of these opposite selective forces revealed that selection at the intrapatient level can promote either T-cell epitope conservation or escape. We predict greater conservation for epitopes contributing significantly to total immune activation levels (immunodominance), and when T_H_ cell infection is concomitant to epitope recognition (trans-infection). We suggest that HIV-driven immune activation in the lymph nodes during the chronic stage of the disease may offer a favorable scenario for epitope conservation. Our results also support the view that some pathogens draw benefits from the immune response and suggest that vaccination strategies based on conserved T_H_ epitopes may be counterproductive.

## Abstract 2

HIV, unlike other viruses, may benefit from immune recognition by preserving the sequence of its T cell epitopes, thereby enhancing transmission between cells.

## Author Summary

A key component of the immune response against viruses and other pathogens is the recognition of short foreign protein sequences called epitopes. However, viruses can escape the immune system by mutating, so epitopes should accumulate high levels of genetic variability. This has been documented in several human viruses, but in HIV, unexpectedly, epitopes tend to be relatively conserved. Here, we propose that this is a consequence of the peculiar interactions that occur between HIV and the immune system. As with other viruses, recognition of HIV epitopes promotes the activation of cytotoxic and helper T lymphocytes, which then orchestrate a cellular immune response. However, HIV infects helper T lymphocytes as their target cell in the body and does so more efficiently when these cells have been activated to participate in an immune response. Mathematical modeling showed that, in some cases, HIV may take advantage of immune activation, thus favoring epitope conservation. This should be more likely to occur with epitopes that trigger more vigorous T-cell responses, and during the process known as “*trans*-infection,” in which helper T lymphocytes are infected while being activated. Our results highlight the potential advantages of an HIV vaccination strategy based on epitopes that stimulate cytotoxic T lymphocytes without specifically stimulating helper T lymphocytes.

## Introduction

Host cellular immunity is thought to be a major factor determining the evolution of HIV-1 and other human viruses, from the intrapatient to the global population level [1],[2]. It has been shown that cytotoxic CD8+ T lymphocytes (CTLs) and helper CD4+ T lymphocytes (T_H_ cells) play a critical role in the early and long-term containment of the virus [3]–[10]. Furthermore, there is epidemiological evidence showing that certain HLA class I alleles (e.g., B27 andB57) influence the rate of disease progression [11]–[14], and HLA-associated HIV-1 polymorphisms may contribute significantly to the global viral diversity and evolution [1],[15]–[19]. Given this, HIV-1 genome regions encoding T-cell epitopes should be under frequent positive or diversifying selection, and thus, these regions should show increased genetic variability. However, unexpectedly, the opposite pattern has been observed in several studies [4],[20]–[27].

The reasons underlying the relatively low genetic diversity of T-cell epitopes in HIV-1 remain poorly understood. One proposed explanation is epitope detection bias [23],[28], whereby mismatches between the peptides used in epitope screening studies and the actual sequence of the assayed viruses tend to produce false negative results in highly variable regions of the viral genome, creating an artificial negative association between immunogenicity and variability. It has also been suggested that epitope conservation may be determined by host factors. The immuno-proteasome preferentially processes hydrophobic residues, and these should tend to show relatively low variability because they often occupy internal regions of the protein that are important for correct folding [29],[30]. Finally, it has been suggested that regions of the viral genome where functional constraint is weaker may have evolved generalized immune escape at the global host population level and thus show fewer extant epitopes than other, more constrained regions [23],[31]. However, analysis of HIV-1 sequences spanning several decades was not consistent with this hypothesis [32], and there is little phylogenetic evidence supporting global escape in HIV-1 [33]. Furthermore, all the above hypotheses fail to explain why no systematic T-cell epitope conservation has been observed in other highly variable and prevalent human viruses such as influenza, hepatitis C, and dengue viruses [34]–[37].

Here, we first carried out a sequence variability analysis to validate and further characterize epitope conservation in HIV-1. Confirming previous findings, sites in the viral genome mapping to both T_H_ and CTL epitopes were consistently less variable than those not mapping to any described T-cell epitopes. In contrast, T-cell epitopes tended to be associated with increased variability levels when this same analysis was carried out for hepatitis C virus (HCV). We also found that HIV-1 epitope conservation was probably determined by intrapatient evolutionary processes and was evident in Gag p24 and Nef proteins even after accounting for epitope detection bias. Based on this, we hypothesized that T-cell epitope conservation may result from the particular interactions established between HIV-1 and the immune system. Although epitope recognition triggers an anti-HIV immune response, the virus replicates more efficiently in activated T_H_ cells [38]–[46]. Therefore, the variability of T-cell epitopes may be determined by the balance between two opposite selective pressures, one favoring immune escape and another favoring immune activation. To tackle this issue, we developed a mathematical model of the intrahost infection dynamics and T-cell responses. We found that sequence conservation may be favored at T_H_ epitopes or CTL epitopes co-mapping with T_H_ epitopes, whereas immune escape should be selected otherwise. The model suggested that epitopes triggering vigorous (immunodominant) T_H_ -cell responses should be more conserved than those triggering weak or moderate responses. This is consistent with the fact that epitope conservation was better supported for highly immunogenic proteins such as Gag p24 and Nef [10],[20]. Furthermore, we predict that epitope conservation may be favored if T_H_ cells frequently become infected in the process of being activated by professional antigen-presenting cells (pAPCs) (transinfection). Since transinfection appears to be an important mechanism for viral dissemination in the lymph nodes during the chronic stage of the disease [47]–[49], our model may help to explain why escape rates tend to slow down as the infection progresses [50]–[52]. Finally, our findings suggest that vaccines that do not elicit HIV-specific T_H_ cell activation may have improved efficacy.

### Empirical Evidence for Epitope Conservation in HIV-1

**Figure 1:**
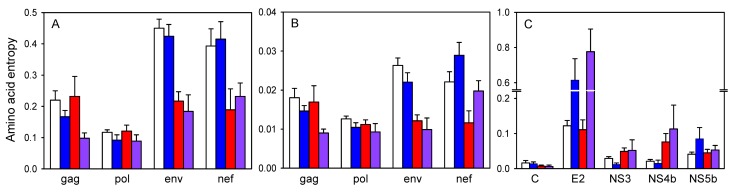
Association between amino acid variability and T-cell epitopes in subtype B HIV-1 (A, B) and HCV 1a (C). Mean ± SEM entropy (*H*) is shown for sites not mapping to any T-cell epitopes (white) and for those mapping to T_H_ epitopes (blue), CTL epitopes (red), or both (purple). In (A) and (C) amino acid entropy was quantified at the host population level (100 sequences from different patients), whereas in (B) it was quantified at the intrapatient level (average from 100 patients containing ≥10 sequences each). For HIV, only Gag, Pol, Env, and Nef are shown because they contain the vast majority of T-cell epitopes. No significant differences in variability associated with T-cell epitopes were found in other genes. Regions with overlapping reading frames were excluded from the analysis. For HCV, only genes with at least five sites in each category were plotted. Notice that the *y*-axis is broken to accommodate the extremely variable epitopes in E2.

To confirm widespread T-cell epitope conservation in HIV-1, we downloaded 100 full-length subtype B sequences from different patients and 220 experimentally validated epitopes (CTL or T_H_) from the Los Alamos HIV-1 database. The epitope list included the “A list” of 88 best-defined epitopes CTL epitopes and also 132 T_H_ epitopes. Using Shannon's entropy (*H*) to quantify variability at each amino acid site, we found that sites mapping to T-cell epitopes tended to be more conserved than those not mapping to any of these epitopes (Figure 1A). This association appeared to be mainly driven by CTL epitopes in Env (two-way ANOVA: *p*<0.001) and Nef (*p*<0.001) proteins, and by T_H_ epitopes in Gag (*p* = 0.005). However, the separate effects of T_H_ and CTL epitopes are difficult to ascertain because they tend to co-map in the HIV genome (Fisher's exact test: *p*<0.001) [53] and, also, because epitopes currently classified as CTL-only may actually be T_H_ epitopes as well, since the latter group has been less extensively studied. The most consistent conservation pattern was observed when comparing sites that mapped to both CTL and T_H_ epitopes (*H* = 0.146±0.016) with those not mapping to any of these epitopes (*H* = 0.255±0.01; nested ANOVA: *p*<0.001).

To check that the results were not dependent on how epitopes have been curated, we repeated the analysis using the complete list of 741 CTL epitopes instead of the “A list.” This confirmed T-cell epitope conservation throughout the genome (nested ANOVA: *p*<0.001). Although the above analyses accounted for differences in variability across genes, we further checked whether epitope conservation may be a by-product of other selective factors in two ways. First, we included RNA structure in the analysis, a major factor constraining HIV variability [27],[54]. We found that nucleotide sites mapping to T-cell epitopes were more conserved than those not mapping to these epitopes regardless of whether they were involved in establishing base-pairs in the genomic RNA structure of the virus (nested ANOVA: *p*<0.001). Second, we verified that epitope conservation was not a byproduct of 5′→3′ variability gradients by introducing genome position as a covariate in the analysis.

To assess whether epitope conservation is determined by intrapatient or host population-level evolutionary processes, we downloaded ≥10 HIV-1 subtype B sequences from each of 100 patients and calculated the average intrapatient amino acid entropy at each amino acid site. Since HIV transmission typically involves one or a few viral particles [55]–[57], the intrapatient sequence entropy largely reflects the variability accumulated over the course of an individual infection. We again observed that sites mapping to T-cell epitopes (CTL “A list” and T_H_) tended to be more conserved (*H* = 0.012±0.001) than those not mapping to any of these epitopes (*H* = 0.018±0.001; nested ANOVA: *p*<0.001; Figure 1B). Indeed, changes in entropy associated with the presence of T-cell epitopes were qualitatively very similar to those observed at the host population level (Figure 1A versus 1B). This suggests that T-cell epitope conservation in HIV-1 is determined at the intra-patient level.

If T-cell epitope conservation was a methodological artifact (e.g., epitope detection bias) or produced by host factors (e.g., selective peptide processing), it should also be evident in other highly variable human viruses. HCV provides a convenient test case because, similar to HIV, it is a rapidly evolving pandemic virus, establishes chronic infections in humans, and is strongly targeted by T-cell immunity [58]–[63]. We aligned 100 HCV subtype 1a polyprotein sequences, calculated the per-site amino acid entropy as above, and downloaded experimentally defined HCV 1a T_H_ or CTL epitopes from the Immune Epitope Database (IEDB). We found that, throughout the genome, amino acid sites mapping to at least one T-cell epitope were significantly more variable (*H* = 0.730±0.007) than those not mapping to any of these epitopes (*H* = 0.636±0.004; nested ANOVA: *p*<0.001), the association being most evident for genes E2 and NS4b (Figure 1C). This pattern contrasts with the results obtained for HIV.

**Figure 2:**
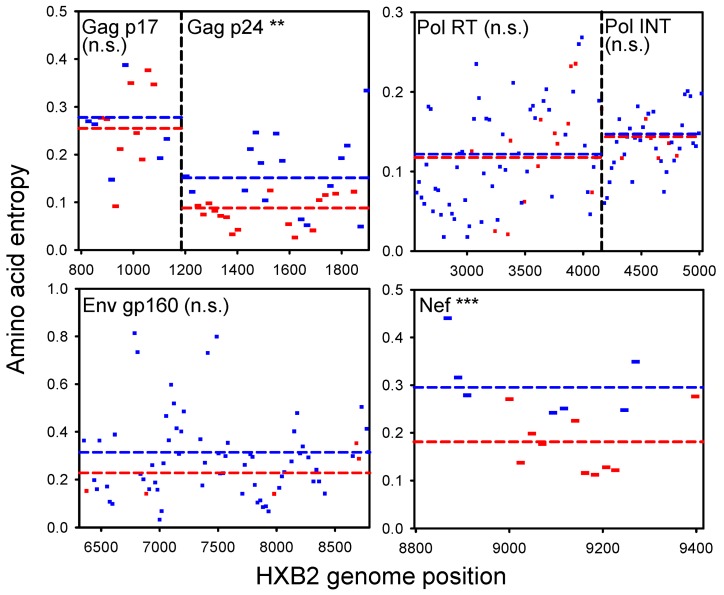
Association between amino acid variability and T-cell immunogenicity in HIV-1 subtype C using data from a high-throughput study [**18**], controlling for epitope detection bias (see text). The average entropy (*H*) is shown for peptides that produced at least one positive immune reaction (red) versus those showing no reactivity (blue). Only genes with at least five peptides in each category are shown. Genome regions with overlapping reading frames were excluded. Dotted lines indicate ANOVA-estimated marginal means. ** 0.001<*p*<0.01; *** *p*<0.001. n.s., not significant.

To further characterize T-cell epitope conservation in HIV, we used a dataset from a high-throughput study in which T-cell responses were determined for a large number of individuals infected with HIV-1 subtype C using the IFNγ enzyme-linked immunospot assay [18]. Thus, epitopes were empirically verified for each patient. These assays involved a battery of synthetic peptides evenly distributed throughout the viral genome, thus eliminating potential problems of region oversampling. Furthermore, the full genome sequence of the infecting virus was available for 113 patients, allowing us to identify every mismatch between the assay peptides and the viral sequence and, thus, to systematically discard epitope detection bias. Among these 113 patients, peptides showing at least one positive immune response were less variable (*H* = 0.165±0.014) than nonimmunogenic peptides (*H* = 0.210±0.008; one-way ANOVA: *p* = 0.005), thus confirming epitope conservation. This difference was significant for Gag p24 (one-way ANOVA: *p* = 0.001) and Nef (one-way ANOVA: *p*<0.001), whereas it was nonsignificant for Gag p17, Pol, and Env (Figure 2). Qualitatively equivalent results were obtained using the number of amino acid substitutions per codon (*d_N_*) instead of entropy, whereas we found no association between immunogenicity and the number of synonymous substitutions (*d_S_*) (Table 1). The latter lack of association further shows that epitope conservation is unlikely to stem from selective pressures acting on RNA structure or from 5′→3′ conservation gradients. Consistently, nonimmunogenic peptides were richer in positively selected codons (*d_N_*/*d_S_*>1) than immunogenic peptides in both Gag p24 and Nef. Finally, we note that these results are probably more reliable for Gag p24 than for Nef since they are based on a larger number of assays (1,485 versus 357).

**Table 1:** Estimated number of nonsynonymous substitutions per codon (*d_N_*), synonymous substitutions per codon (*d_S_*), and percentage of codons under positive selection (% *d_N_>d_S_*) in peptides showing at least one positive immune reaction (epitopes) versus those showing no reactions (non-epitopes), using data from a HIV-1 subtype C high-throughput study [18].

Protein	Epitopes			Nonepitopes		
	*d_N_*	*d_S_*	% *d_N_>d_S_*	*d_N_*	*d_S_*	% *d_N_>d_S_*
Gag p17	2.8±0.3	4.4±0.2*	6.5±2.4	2.8±0.4	5.4±0.3*	6.2±3.7
Gag p24	0.8±0.1**	3.9±0.3	1.4±0.9**	1.5±0.1**	4.2±0.3	5.8±1.0**
Pol RT	1.1±0.1	4.9±0.3	1.8±1.1	1.2±0.1	4.6±0.1	3.3±0.5
Pol INT	0.8±0.1	3.8±0.5	2.1±1.4	0.8±0.1	3.8±0.3	2.2±0.7
Env	2.3±1.3	4.5±1.0	11.3±3.6	3.7±0.4	5.6±0.3	10.3±1.0
Nef	1.9±0.2**	5.1±0.4	2.8±1.2*	3.1±0.2**	5.9±0.4	7.3±1.5*
Total	1.7±0.2*	4.7±0.2	4.4±0.8*	2.2±0.1*	5.0±0.1	6.2±0.4*

* ANOVA comparing epitopes and nonepitopes: 0.01<*p*<0.05.

** 0.001<*p*<0.01.

### The Immune Activation Model

We sought to develop a model that could account for the following observations made above: T-cell epitopes are less variable than other regions of the viral genome; epitope conservation appears to be determined by intrapatient evolutionary processes; after ruling out possible confounders, the conservation signal is found mainly in highly immunogenic proteins; HCV and other human viruses do not show widespread epitope conservation. Also, since both T-cell escape and epitope conservation have been documented in HIV-1, it becomes important to identify key factors determining which of these outcomes should take place.

**Figure 3:**
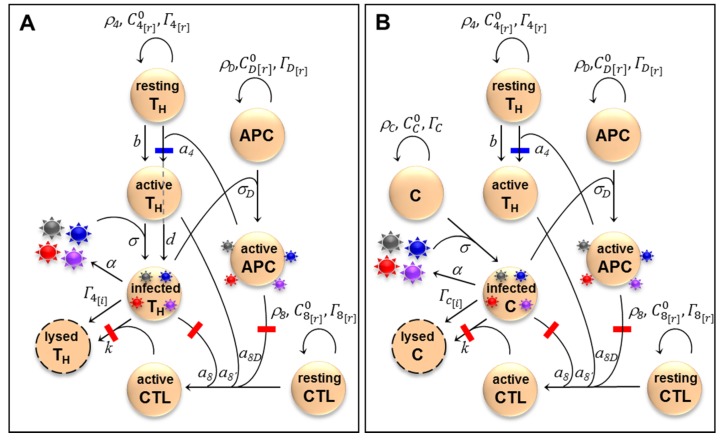
Schematic representation of the HIV immune activation model and the control model. (A) Model of the cellular immune response against HIV. In the absence of immune activation, pools of resting T_H_ (CD4) cells, CTLs (CD8), and pAPCs divide at rates, *ρ_4_*, *ρ_8_*, *ρ_D_* and die at rates 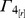, 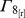, and 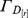, reaching homeostatic concentrations 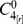, 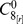, and 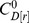, respectively. Activated T_H_ cells become infected through contact with free virions at a rate constant *σ*, whereas resting cells are assumed to be non-susceptible to the virus. T_H_ cells are activated at a rate constant *a_4_* after contacting a pAPC with an HIV epitope or by other antigens at rate constant *b* (background activation). T_H_ cells establishing synapses with pAPCs have a probability *d* of being concomitantly infected with the same viral type. Infected cells release virions at a rate constant *α* and die at a rate constant 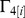. CTL pre-activation occurs after contacting infected cells (*a_8_*) or pAPCs (*a_8D_*). A co-stimulatory signal from activated T_H_ cells is necessary for completing CTL activation (*a_8′_*). Infected cells are lysed by CTLs at a rate constant *k*. Death rate constants for activated T_H_ cells 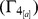, CTLs 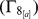, and pAPCs 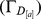 and virion inactivation rates (*Γ_V_*) are not shown for simplicity. The full list of variables and parameters is available in Appendix S1, which also provides references to empirical work justifying the parameter values used (see also main text). A fraction *μ* of the virions released in each cell infection become escape mutants. Avoidance of CTL activation or CTL-mediated killing leads to CTL escape (red bars), whereas avoidance of T_H_ cell activation leads to T_H_ escape (blue bars). The model allows full T-cell (purple), T_H_-only (blue), and CTL-only (red) escape mutants. (B) Control model in which the virus targets a nonimmune cell type C (e.g., hepatocytes, epithelial cells, etc.) instead of T_H_ cells. Two key differences with the HIV model are that viral replication is not dependent upon immune activation and that transinfection does not take place. Variables, parameters, and equations for this model are also shown in Appendix S1.

We suggest that, since HIV-1 replicates more efficiently in activated T_H_ cells, epitope conservation may provide payoffs to the virus by increasing the pool of virus-susceptible cells. On the other hand, epitope conservation is costly because it triggers an anti-HIV CTL response. To explore how the complex interactions established between HIV-1 and the cellular immune system may favor or select against epitope escape, we built a mathematical model involving T_H_ cells, CTLs, and pAPCs (Figure 3A). We included pAPCs because T_H_ cell activation is mediated by MHC II epitopes, which are only present in pAPCs. Also, pAPCs are an important viral transmission vehicle (see below). In the model, T_H_ cells could be activated by HIV epitopes or other, non-HIV, antigens (e.g., from microbial translocation). We denoted the latter background activation. Dendritic cells are the main type of pAPCs in the context of an HIV infection, but macrophages also fall into this category. CTL activation required recognition of an HIV epitope presented by the MHC I of an infected T_H_ cell or a pAPC, and co-stimulatory cytokines released from active T_H_ cells. Cytokine co-stimulation was not epitope-specific, meaning that it could also come from background-activated T_H_ cells. Background activation of CTLs is not relevant in the context of the model and was thus not considered. Activated CTLs lysed infected T_H_ cells after recognizing an MHC I epitope. However, error-prone replication gave rise to progeny virions carrying escape mutations in their CTL or T_H_ epitopes. We defined CTL escape mutants as those failing to trigger MHC I-mediated CTL activation and cell killing, T_H_ escape mutants as those failing to trigger MHC II-mediated T_H_ cell activation, and T-cell escape mutants as those escaping both types of response.

Critically, HIV-1 replication and viral load are dependent on levels of T_H_ cell activation [38]–[41]. In nonactivated cells, the efficiency of reverse transcription is lower than in activated cells because of the limited dideoxynucleotide availability , low ATP levels hamper nuclear transport of the pre-integration complex, and gene expression is less well-supported by key transcription factors such as NF-κB and NFAT [42]–[44]. As a result, the infection cycle can be arrested at the reverse transcription step and the incomplete viral DNA degraded unless the cell undergoes activation within days following viral entry [42],[45],[46]. T_H_ cells can become infected in the process of being activated by pAPCs, though. A mechanism for this is transinfection, whereby dendritic cells can transmit virions bound to their DC-SIGN or L-SIGN lectins to the T_H_ cells with which they establish MHC II-type immune synapses [48],[49]. Another possible mechanism is cis-infection, whereby virions released from infected dendritic cells or macrophages are transmitted to synapsing T_H_ cells. However, pAPCs are not major viral producers [64], and therefore, we neglected viral replication in these cells and cis-infection for simplicity. The contribution of HIV-specific immune activation and transinfection to viral spread and pathogenesis is supported by the observation that HIV-specific T_H_ cells are more readily infected than other subpopulations of active T_H_ cells [65], reducing their life span and compromising the generation of effective anti-HIV responses [66].

We also built a “control” model in which the virus infects nonimmune cells (denoted C) instead of T_H_ cells, but which was otherwise identical to the HIV model (Figure 3B). This allowed us to parallel the comparison between HIV and HCV made above (Figure 1). To address whether T_H_ and CTL escape mutants should be capable of outgrowing the wild-type virus given the intrapatient selective forces imposed by the cellular immune response in the HIV and control models, we considered a single immune response-escape cycle. Successive cycles ultimately leading to immune exhaustion and AIDS have been modeled previously and are important for understanding the natural history of the infection and pathogenesis [67]–[69].

Some of the model parameter values could be adjusted based on available empirical evidence. We chose a cell division rate (*ρ*) of 0.05 day^−1^ (i.e., a doubling time of 13.9 days) and a death rate 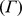 of 0.005 day^−1^ for resting cells (a half-life of t_1/2 = _139 days) and of 0.1 day^−1^ (t_1/2 = _6.9 days) for activated cells [70]. The homeostatic T_H_ cells concentration was 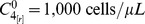 [71]. We assumed that cellular division was suppressed above the homeostatic value, and this was modeled using a unit step function (denoted Θ). The death rate constant of infected cells was 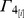 = 1 day^−1^ [72]–[77], and the viral production rate of infected cells (*α*) was chosen such that the burst size was *α*/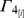 = 5,000 particles/cell, a value which falls within the realistic range of 1×10^3^–5×10^4^ particles/cell [78]–[81]. The rate constant for CTL-mediated cell killing was *k* = 0.1 day^−1^ = 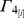/10, such that at equilibrium approximately 10% of virus-induced cell death was due to CTL activity [52]. Published virion clearance constants (*Γ*_V_) vary amply, from 0.3 day^−1^ [72],[76] to >30 day^−1^ [82], and we chose an intermediate value of 15 day^−1^. The HIV-1 mutation rate is approximately *μ* = 3×10^−5^ per nucleotide site per cell infection [83]. To consider a single escape mutant, we set the mutation rate to *μ* = 10^−5^. For some parameters, such as the in vivo rate of viral absorption to T_H_ cells (*σ*) and pAPCs (*σ_D_*), we did not find empirical data and these were adjusted to produce realistic peak titers, set-point titers, and fractions of infected cells. Virus–cell and cell–cell interactions were modeled as AB/(A+B), where A and B are the interacting elements. For instance, T_H_ cells infected with the wild-type virus 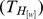 were killed by activated CTLs 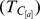 at a rate 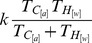. In this function, cell killing is limited by target cell availability if 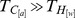 and by CTL availability if 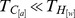. Thus, cell infection and activation were modeled as saturating processes. We assumed that infection rates for each viral type saturate as a function of the total viral density. For instance, activated T_H_ cells 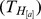 were infected by wild-type virions (*V_w_*) at a rate 
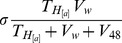
, where 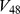 is the load of T-cell escape virions. Conversely, cells were infected by escape virions at rate 
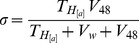
. Thus, wild-type infection rates decreased as the density of escape viruses increased and vice versa, reflecting competition among viruses for cells. The full list of variables and parameters and the systems of ordinary differential equations defining the models are shown in the Appendix S1.

**Figure 4:**
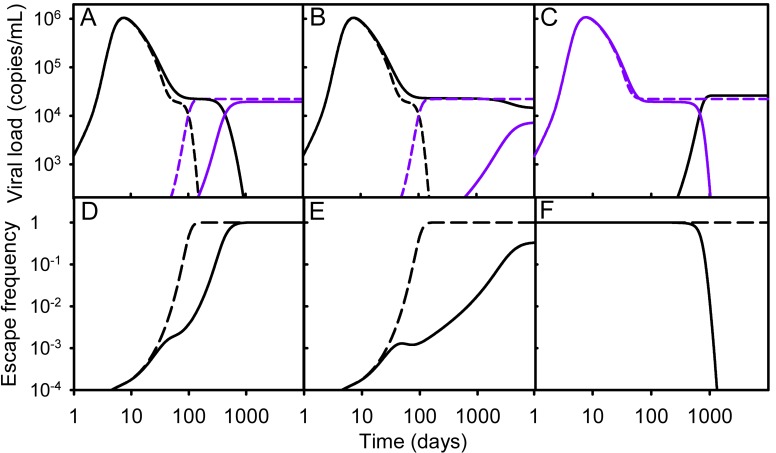
Simulated viral load versus time for combinations of parameter values producing biologically meaningful peak loads, viral loads at set point (copies/mL), and escape rates (days^−1^). In top panels, wild-type and T-cell (CTL/T_H_) escape variants are shown in black and purple, respectively. Solid lines refer to the HIV model, whereas dashed lines correspond to the control model. HIV-specific activation rate constants (*a_4_*, *a_8_*, *a_8D_*, *a_8′_*), the background activation rate constant (*b*), and transinfection probability (*d*) were as indicated below, whereas all other parameters values were set as indicated in the text. (A) *a_4_* = *a_8_* = *a_8D_* = *a_8′_* = 0.1, *b* = 0.001, *d* = 0.03; (B) *a_4_* = *a_8_* = *a_8D_* = *a_8′_* = 0.1, *b* = 0.001, and *d* = 0.037; (C) *a_4_* = *a_8_* = *a_8D_* = *a_8′_* = 0.15, *b* = 0.001, *d* = 0.03, and the infection was started with a T-cell escape mutant. (D, E, and F) Changes in the intrapatient frequency of the escape mutant for the HIV (solid) and control (dashed) models obtained in (A), (B), and (C), respectively. (D) The calculated rate of escape, as defined in previous work [50], was 0.012 day^−1^ in the HIV model and 0.091 day^−1^ in the control model. (E) The escape mutant reached a stationary frequency of 0.324 in the HIV model and became fixed in the control model (fixation rate: 0.091 day^−1^). (F) The escape mutant was selected against and reverted to the wild-type in the HIV case (reversion rate: 0.012 day^−1^), whereas it remained fixed in the control model.

We started simulations with one infected cell and homeostatic values of resting target cells (T_H_ or C). We also provided a large pool of HIV-susceptible active T_H_ cells for the primary infection to mimic the initial spread of HIV through the mucosa or gut-associated lymphoid tissue (GALT). The model captured the typical HIV infection dynamics, in which viral load increases rapidly until reaching a peak days or weeks after transmission and subsequent exhaustion of the initial pool of susceptible cells and CTL activation make the viral load drop but fail to eradicate the infection (Figure 4). A dynamic equilibrium or set point was reached in which the virus continued to replicate, the immune system remained activated, and viral loads showed stable values within the typical range of 10^3^ to 10^5^ viral copies/mL [84] for the parameter values used. The control model produced similar dynamics.

CTL (non-T_H_) escape mutants always became dominant in both the HIV and control models, whereas T_H_ (non-CTL) cell escape mutants were neutral in the control model and neutral or deleterious in the HIV model (not shown). The reason why T_H_ cell escape does not per se provide a fitness advantage is that these mutants can be targeted by CTLs activated by the wild-type virus or other antigens. In addition, in the HIV case, T_H_ escape mutants act as a sink of susceptible cells and are thus dependent on cells activated by the wild-type virus or on background activation for replicating, making them potentially deleterious. Therefore, considering CTLs and T_H_ cells together, full T-cell (T_H_ and CTL) escape may be favored or selected against depending on the balance between the benefits of CTL escape and the potential costs of T_H_ escape. We are able to find parameter values that produced T-cell escape rates similar to those reported in studies of patient serial samples (Figure 4) [50],[67]. The timing of escape could also be varied from weeks postinoculation to years. In contrast, other parameter combinations disfavored T-cell escape mutants, and epitopes remained invariant if the infection was initiated with the wild-type, or they reverted to the wild-type if the infection was initiated with escape mutants (Figure 4C, F). In these cases, epitope conservation was promoted. In contrast, in the control model, T-cell escape occurred systematically and the rate of escape was faster than in the HIV case for the same parameter values. This showed that epitope conservation can be explained by the particular nature of the HIV infection, in which T_H_ escape can be costly for the virus.

**Figure 5:**
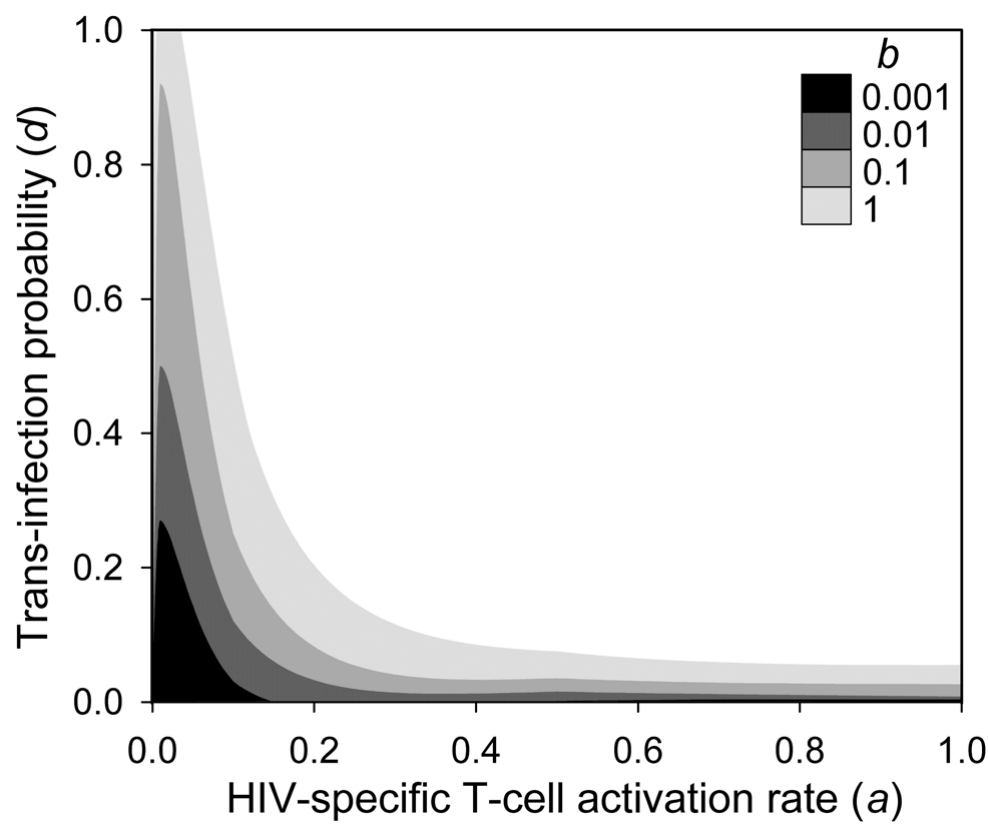
Immune escape dependence on the HIV-driven T-cell activation rate constant (*a_4_*), trans-infection probability (*d*), and background activation (*b*). Shaded areas indicate parameter combinations for which T-cell escape mutants became dominant (frequency >0.5) after *t* = 1000 iterations (days). T-cell activation rate constants were set equal to one another (*a_8_* = *a_8D_* = *a_8′_* = *a_4_*), and the rest of parameter values were as indicated in the text.

A central goal of our HIV model was to identify factors determining whether T-cell escape or epitope conservation should take place. We found that transinfection probability and immune activation levels were two such factors (Figure 5). Since transinfection implies a temporal and spatial association between T_H_ cell activation and infection, there should be some correlation between the type of epitope (wild-type or escape mutant) presented by a pAPC and the type of virion transmitted to synapsing T_H_ cells. We denoted this correlation *d*. In the absence of transinfection or if every pAPC contained equal amounts of wild-type and T-cell escape virions, then *d* = 0, and therefore, T_H_ cells activated by the wild-type virus would be fully accessible to T-cell escape viruses. As a result, T_H_ escape should not be detrimental to the virus, and considering the benefit of evading CTLs, the net effect of T-cell escape should be positive. In the control model, since there was no possible transinfection, T-cell escape was always advantageous. In the HIV model, in contrast, as *d* increased, T-cell escape mutants had less and less access to T_H_ cells activated by the wild-type virus, and since they could not produce their own pools of activated T_H_ cells, these mutants had a selective fitness disadvantage and failed to outgrow the wild-type virus. The magnitude of this disadvantage depended inversely on levels of background activation, because the latter is a source of susceptible cells equally accessible to the wild-type and the escape mutant. Also, for *d*>0, the outcome depended on the strength of epitope-specific immune activation relative to background activation. If the epitope failed to produce T-cell activation (anergy), there was obviously no advantage associated with escape. Simulations also showed, however, that if the epitope triggered a strong T-cell activation (immunodominance), the escape mutant was disfavored too because the pool of activated T_H_ cells to which the escape mutant had limited access represented a large portion of total susceptible cells. Therefore, the model suggests that, in HIV, immune escape should preferentially take place among epitopes triggering weak to moderate T-cell responses.

### Conclusions

If immune avoidance is beneficial for a virus, escape mutants should tend to accumulate throughout the course of the infection unless they incur fitness costs (i.e., defects in other steps of the infection cycle not related to immune evasion) exceeding the benefits of escape. According to this, fast mutating viruses eliciting strong T-cell responses and establishing chronic infections should show the highest frequencies and rates of escape. Upon transmission to new hosts with different HLA types, however, the selective advantage of these mutants disappears and the virus should tend to revert to the wild-type if the escape mutation has some fitness cost, as has been amply documented in HIV [85]–[91]. This indeed constitutes a particular instance of antagonistic pleiotropy, a frequent process among RNA viruses whereby selectively advantageous mutations in one environment become deleterious in other environments [92]. As a result of this alternating selective regime, viral sequence variability at the population level should be promoted and epitopes should tend to be more variable than other genome regions. Previous work and our sequence data analysis (Figure 1) show that HCV fits well into this pattern, whereas HIV-1 does not.

The immune activation model provides a possible explanation for the unexpected epitope conservation in HIV-1. The model predicts that T-cell escape can be selected against depending on factors such as transinfection probability, immune activation levels, or epitope strength. Although CTL escape should per se be advantageous for the virus, CTL epitopes may be conserved if they co-map with T_H_ epitopes. As shown here and in previous work [53], CTL/T_H_ epitope co-mapping occurs more often than expected by chance, probably because these two types of epitopes share common cellular pathways. Depending on the above factors, thus, T-cell escape may occur only in some genome regions or only in certain individuals. In this sense, the model contributes to resolving the apparent paradox between T-cell epitope conservation and the large body of evidence showing that T-cell immunity is an important selective factor promoting HIV variability. These disparate findings are unlikely to result from use of different datasets or methodologies. For instance, in one study, it was found that that there was a general positive association between specific HLA types and the occurrence of escape mutations, but that this association was negative in some cases (i.e., epitopes were significantly more conserved among patients with the relevant HLA type than among those with nonmatching HLAs) [93]. It is also noteworthy that some escape mutations are rapidly favored, whereas others become dominant only after years of intrahost replication [52],[94]. This is often interpreted in terms of the fitness costs of escape mutations [51],[67],[88]–[90]. However, the immune activation model can also account for variable rates of escape even in the absence of fitness costs. Indeed, our model did not assume any fitness costs for escape mutations. Such costs may explain why some escape mutations increase in frequency more slowly than others, fail to be selected, or do not spread in the host population [26]. However, they cannot explain why genome regions containing T-cell epitopes tend to be more conserved than those not containing epitopes, since costs should equally apply to both.

The combination of parameters for which we predicted T-cell epitope conservation should be more likely in the lymph nodes at the chronic stage of the disease, during which HIV-specific activation of T_H_ cells contributes to sustaining the infection and pAPC-mediated coupled activation-infection of T_H_ cells should be frequent [47]–[49]. In contrast, the GALT and other mucosa contain large pools of background-activated and recent memory T_H_ cells which are exploited by HIV during primary infection, thus making the virus less dependent on its own ability to activate T_H_ cells [95]–[97]. It has been previously shown that T-cell escape rates are higher during primary infection than in the chronic stage of the disease [50]–[52]. Again, this is often interpreted in terms of fitness costs, since escape mutations paying weak fitness costs should be selected faster and be detected at earlier disease stages than those paying strong costs. Another interpretation is that epitopes triggering more vigorous T-cell responses tend to experience faster escape due to the stronger selective pressure exerted. Our model offers yet another possible interpretation: T-cell escape may actually be slowed down whenever HIV depends on its own ability to activate T_H_ cells for replicating, and this is more likely to occur during the chronic stage than during primary infection. If the timing of escape was determined by fitness costs, then late escapes should tend to revert faster than early escapes upon transmission to new individuals with different HLA types because of their greater deleteriousness, whereas if the timing of escape was determined by immune activation levels, the reverse should be true. These predictions offer a way of testing the above alternative explanations for why rates of escape differ during the primary and chronic stages of the disease. Sequence analysis revealed that, after accounting for dataset biases, epitope conservation occurred mainly in Gag p24 and Nef (Figure 2). As expected from the immune activation hypothesis, these proteins contain several immunodominant epitopes [20],[98], whereas they are not necessarily more likely to exhibit fitness costs than other HIV genes.

Finally, our model suggests that vaccines based on conserved T_H_ epitopes might be counterproductive. By creating a pool of HIV-responsive T_H_ cells, they may pave the way for viral replication in certain body compartments. This might contribute to understanding the unexpected results of the STEP vaccine trial, in which the vaccinated group was found to be at higher risk of infection than the placebo-treated control group, although there are many other possible explanations [99],[100]. According to our model, an efficient approach to HIV vaccination may be to use CTL epitopes that do not stimulate T_H_ cells. These epitopes may be combined with non-HIV T_H_ epitopes that would co-stimulate CTLs without providing the immune system with a pool of HIV-specific memory T_H_ cells. The idea that immune activation can favor pathogen replication and that, consequently, vaccines based on conserved epitopes may be counterproductive has also been proposed for *Mycobacterium tuberculosis* [101], although the mechanisms at play have not been elucidated in this case and may potentially differ from those of HIV. Interestingly, tuberculosis-specific T_H_ cells are also preferentially depleted in HIV-infected individuals, whereas this is not observed in cytomegalovirus, another opportunistic pathogen [102],[103]. It is possible that, by triggering immune activation, *M. tuberculosis* may benefit from HIV-mediated depletion of T_H_ cells and subsequent immune impairment.

## Methods

### Sequence Analysis

A BLAST of the entire reference subtype B sequence (HXB2) was performed using the HIV Sequence Database search tool (www.hiv.lanl.gov/components/sequence/HIV/search/search.html), restrictfing the search to one sequence per patient. For each Gag, Pol, Env, Vif, Vpu, Vpr, and Nef, 100 translated sequences were aligned using the MUSCLE algorithm implemented in MEGA v5 (megasoftware.net) and HXB2 as reference sequence. Sequences with premature stop codons or partial readings were removed. Protein Shannon's entropy was calculated for each site of the alignment as 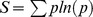, where *p* denotes the frequency of each of the amino acids present at this site. Gaps were treated as another amino acid. These calculations were carried out using Entropy-one tool of the HIV Sequence Database with default options. To estimate synonymous (*d_S_*) and nonsynonymous (*d_N_*) substitutions rates from nucleotide sequences, we first used the Datamonkey server (www.datamonkey.org) to select the best substitution model and to identify significant recombination breakpoints using the GARD algorithm [104], using default parameters except for the inferred substitution model. Using this output, we run the SLAC algorithm [105] implemented in the HYPHY package [106] to identify codons under significantly positive or negative selection at a 0.1 probability threshold, and to estimate *d_N_* and *d_S_*. For the intrapatient entropy analysis, HIV-1 subtype B sequences were downloaded for each Gag, Pol, Env, and Nef from the HIV Sequence Database using the intrapatient search tool and restricting the search to patients with at least 10 sequences available and with known time since infection/seroconversion or Fiebig stage. Entropy values were obtained as above and, for each amino acid site, the within-host entropy was averaged over 100 patients. For HCV, 100 full-length subtype 1a genomes were downloaded from GenBank and translated to polyprotein sequences. Alignments and subsequent analyses were done as above, using the H77 sequence as reference.

### Epitope Mapping and Analysis

HIV CTL and T_H_ epitopes were downloaded from the HIV Molecular Immunology Database (www.hiv.lanl.gov/content/immunology/tables/tables.html). For CTL epitopes, we used the full set of 741 entries or a curated list of 88 best defined epitopes (“A list”), whereas for T_H_ epitopes we used the 132 available entries (no curated list has been defined for this group). Each epitope was aligned to the HXB2 sequence, and HXB2 genome sites were classified based on whether they mapped to at least one epitope. HCV subtype 1a epitopes were downloaded from the IEDB (www.immuneepitope.org) selecting the specific epitope type (MHC I for CTL epitopes and MHC II for T_H_ epitopes), peptides from proteins as structure type, and *Homo sapiens* as host organism. This yielded 101 CTL and 27 T_H_ epitopes. Genome-wide differences in amino acid entropy associated with the presence of T-cell epitopes were tested using a fixed-factor nested ANOVA (presence/absence of T-cell epitope nested within gene). For HIV, the contribution of RNA structure to the observed variability was tested using nucleotide instead of amino acid sites and adding this factor to the above ANOVA design (paired/nonpaired site according to published structure, nested within gene). The effect of 5′→3′ gradients was tested by including genome position as a covariate in the model. Separate effects of CTL and T_H_ epitopes were tested using two-way ANOVAs.

### High-Throughput Dataset Analysis

We used data from a study in which 396 synthetic peptides were tested for immunogenicity in a HIV-1 subtype C cohort from South Africa [18]. The dataset is freely available at www.hiv.lanl.gov/content/immunology/hlatem/study3/index.html. We restricted the analysis to 113 individuals for which the full-length genome of the infecting virus was available. Of the total 44,748 assays considered (113 patients×396 peptides), the peptide matched exactly the amino acid sequence of the virus only in 13,127 cases (29.3%). The straightforward correction for epitope detection bias would be to restrict the analysis to this subset. However, 118 of the total 179 positive T-cell reactions (65.9%) corresponded to nonmatching peptides. These positives may be due to cross-reactivity, but given that IFN responses can persist for years [107], they could also correspond to cases of immune escape. Therefore, to avoid missing a significant fraction of immune-driven viral variability, we also included these nonmatching T-cell-positive peptides as valid assays. If any, this should produce an artificially positive association between variability and immunogenicity. We then classified peptides in two categories according to whether or not they produced at least one positive T-cell reaction and tested for differences between these two groups using a one-way ANOVA in which the number of valid assays per peptide was included as a covariate in the model. Amino acid entropy values for each group correspond to marginal means estimated from the ANOVA model.

### Immune Activation Model

We developed a system of ordinary differential equations describing how T_H_ cell, CTL pAPC counts, and viral loads vary with time as described in the text and Figure 3A. We also build a control model including a nonimmune viral target cell type C (Figure 3B). The full list of variables and parameters, a detailed description of the model, and the systems of ordinary differential equations are available in Appendix S1. Simulations were performed in Mathematica 8 (Wolfram Research). SBML files describing the model have been deposited in the BioModels Database (MODEL1302180001 for immune activation model and MODEL1302180002 for control model)-

## Supporting Information

Appendix S1 Model variables, parameters, and systems of differential equations describing the dynamics of wild-type and T-cell (T_H_/CTL) escape viruses for the HIV and control models.
(PDF)

## Abbreviations

CTL: cytotoxic T-lymphocyte
GALT: gut-associated lymphoid tissue
HCV: hepatitis C virus
HIV: human immunodeficiency virus
pAPC: professional antigen-presenting cell
T_H_ cell: CD4^+^ helper T lymphocyte
