# Supplementary material for: Immune Activation Promotes Evolutionary Conservation of T-Cell Epitopes in HIV-1
Source: PLoS Biol. 2013 Apr 2;11(4):e1001523. doi: 10.1371/journal.pbio.1001523 (PMC3614509; doi:10.1371/journal.pbio.1001523)
Supplement: Appendix S1 — Model variables, parameters, and systems of differential equations describing the dynamics of wild-type and T-cell (TH/CTL) escape viruses for the HIV and control models. (PDF) [file pbio.1001523.s001.pdf]

Appendix

List of variables in the models

Variable	Definition
$T_{H[r]}$	Resting T_H cells
$T_{H[a]}$	Activated T_H cells
$T_{H[w]}$	T_H cells infected with the wild-type virus
$T_{H[48]}$	T_H cells infected with the full T-cell (T_H/CTL)-escape mutant
$T_{C[r]}$	Resting CTLs
$T_{C[Pr1]}$	CTLs pre-activated by epitope-presenting cells (infected T_H/C cells or pAPCs)
$T_{C[Pr2]}$	CTLs receiving co-stimulatory cytokines
$T_{C[a]}$	Fully activated CTLs
$D_{[r]}$	Resting pAPCs
$D_{[w]}$	pAPC presenting a wild-type epitope
$D_{[48]}$	pAPC presenting a T-cell (T_H/CTL)-escape epitope
V_W	Load of wild-type virus
V_{48}	Load of T-cell (T_H/CTL) escape virus
$C_{[w]}$	C cells infected with the wild-type virus (control model)
$C_{[48]}$	C cells infected with the T_H/CTL-escape mutant (control model)

List of parameters in the models with values used and references

Parameter	Definition	Values	Refs
ρ_4	Proliferation rate of T_H cells	0.05 day $^{-1}$	[1]
$C^0_{4[r]}$	Homeostatic concentration of resting T_H cells	1000 /μL	[2]
$\Gamma_{4[r]}$	Death rate of resting T_H cells	0.005 day $^{-1}$	[1]
$\Gamma_{4[a]}$	Death rate of activated T_H cells	0.1 day $^{-1}$	[1]
$\Gamma_{4[i]}$	Death rate of infected T_H cells	1 day $^{-1}$	[3–8]
ρ_8	Proliferation rate of CTLs	ρ_4	
$C^0_{8[r]}$	Homeostatic concentration of resting CTLs	3000 /μL	
$\Gamma_{8[r]}$	Death rate of resting CTLs	$\Gamma_{4[r]}$	
$\Gamma_{8[pr]}$	Death rate of pre-activated CTLs	$\Gamma_{4[r]}$	
$\Gamma_{8[a]}$	Death rate of activated CTLs	$\Gamma_{4[a]}$	
ρ_D	Proliferation rate of pAPCs	ρ_4	
$C^0_{D[r]}$	Homeostatic concentration of pAPCs	100 /μL	[9]
$\Gamma_{D[r]}$	Death rate of resting pAPCs	$\Gamma_{4[r]}$	
$\Gamma_{D[a]}$	Death rate of epitope-presenting pAPCS	$\Gamma_{4[a]}$	
ρ_C	Proliferation rate of C cells (control model)	ρ_4	
C^0_C	Homeostatic concentration of C cells (control model)	set-point $T_{H[a]}$	
Γ_C	Death rate of C cells (control model)	$\Gamma_{4[a]}$	
$\Gamma_{C[i]}$	Death rate of infected C cells (control model)	$\Gamma_{4[w]}$	
a_4	pAPC-mediated rate of T_H cell activation	0-1 day $^{-1}$	
$a_{8'}$	Pre-activation rate of CTLs receiving co-stimulatory cytokines	a_4	
a_8	Pre-activation rate of CTLs by infected T_H (C) cells	a_4	
a_{8D}	Pre-activation rate of CTLs by pAPCs	a_4	
b	Fraction of resting T_H cells undergoing background activation	0-1 day $^{-1}$	
d	Trans-infection probability	0-1	
k	CTL-mediated rate of infected cell killing	0.1 day $^{-1}$	[10]
σ	Viral infectivity	1 day $^{-1}$	
σ_D	Rate of virion absorption by pAPCs	1 day $^{-1}$	
α	Virus production rate in infected cells	5000 $\Gamma_{4[i]}$ day $^{-1}$	[11–14]
Γ_V	Inactivation rate of free virions	15 day $^{-1}$	[3,7,15]
μ	Mutation rate to escape virus	10^{-5}	[16]

HIV immune activation model

Description

Resting T_H cells replicate with rate constant ρ_4 if their concentration is below an homeostatic goal $C^0_{4_{[r]}}$ and do not replicate otherwise (modeled with a step function Θ). The homeostatic goal applies to resting cells only and that, therefore, the number of activated T_H is not limited, allowing for clonal expansion. Resting and activated T_H cells die at rates $\Gamma_{4_{[r]}}$ and $\Gamma_{4_{[a]}}$, respectively.

Background activation of T_H cells occurs at a rate $bT_{H[r]}$, and HIV-specific activation occurs at a rate $T_{H[r]}\left(\frac{D_{[w]}}{T_{H[r]}+D_{[w]}}\right)$ when resting T_H cells contact pAPCs presenting the wild-type epitope. Escape mutants do not trigger T_H cell activation. The activation rate depends on the relative abundance of each cell type. For instance, if pAPCs are rare compared to resting T_H cells, the rate of T_H cell activation is essentially limited by pAPC availability. This type of expression is used for all cell-cell and virus-cell interactions in the model.

Of the total number of activated cells, a fraction *d* becomes concomitantly infected with the wild-type virus and a fraction 1 – *d* is not infected but becomes susceptible to infection with the wild-type or the escape mutant. Activated T_H cells are infected at a rate $\sigma T_{H[a]}\left(\frac{V_w+V_{48}}{T_{H[a]}+V_w+V_{48}}\right)$. As above, infection rates depend on the relative abundance of each virus and cell. When virions are more abundant than target cells, cells become a limiting resource. When cellular resources are limited the infection rate of the wild-type decreases as the escape mutant becomes more abundant, and vice-versa. Therefore, there is competition among viruses for cells.

Infected cells are killed by the wild-type virus and escape mutant at rates $\Gamma_{4_{[i]}}T_{H[w]}$ and $\Gamma_{4_{[i]}}T_{H[48]}$, respectively. In addition, cells infected by the wild-type virus are lysed by activated CTLs at a rate $kT_{C[a]}\left(\frac{T_{H[w]}}{T_{C[a]}+T_{H[w]}}\right)$, whereas cells infected with the escape mutant are not lysed by CTLs.

Resting CTLs replicate with rate constant ρ_8 if their concentration is below their homeostatic goal (i.e. if $C^0_{8_{[r]}} - T_{C[r]} > 0$) and do not replicate otherwise. They death rate constant is $\Gamma_{8_{[r]}}$. As in the case of T_H cells, the homeostatic goal applies to resting cells only. CTLs become pre-activated (Pr1) by recognizing the wild-type epitope presented by infected cells or pAPCs, at rates $a_8T_{C[r]}\left(\frac{T_{H[w]}}{T_{C[r]}+T_{H[w]}}\right)$ and $a_{8D}T_{C[r]}\left(\frac{D_{[w]}}{T_{C[r]}+D_{[w]}}\right)$, respectively. These pre-activated cells undergo full activation after receiving a co-stimulatory signal from activated (non-infected) T_H cells, which occurs at a rate $a_{8'}T_{C[Pr1]}\left(\frac{T_{H[a]}}{T_{C[Pr1]}+T_{H[a]}}\right)$. Resting CTLs can also

become pre-activated by the co-stimulatory signal (Pr2) and then become fully activated after recognizing the wild-type epitope. Activated CTLs die with rate constant $\Gamma_{8_{[a]}}$.

Resting pAPCs replicate with rate constant ρ_D if their total concentration is below their homeostatic goal, i.e. if $C^0_{D[r]} - D_{[r]} - D_{[w]} - D_{[48]} > 0$, and do not replicate otherwise. Notice that here the homeostatic goal applies to all cells, such that there is no clonal expansion. pAPCs become activated after processing wild-type or escape mutant virions at a rate $\sigma_D D_{[r]}\left(\frac{V_w+V_{48}}{D_{[r]}+V_w+V_{48}}\right)$. Resting and activated pAPCs die with rate constants $\Gamma_{D_{[r]}}$ and $\Gamma_{D_{[a]}}$, respectively.

T_H cells infected with the wild-type virus produce virions at a rate $\alpha T_{H[w]}$. A fraction μ of the viral progeny is constituted by escape mutants and a fraction $(1-\mu)$ by wild-type viruses. T_H cells infected with the escape mutant produce virions at a rate $\alpha T_{H[48]}$. Back mutation from the escape mutant to the wild-type is neglected. Virions are inactivated with rate constant Γ_V.

<u>Equations</u>

$$\frac{d}{dt}T_{H[r]} = \rho_4\Theta\left(C^0_{4_{[r]}} - T_{H[r]}\right) - \Gamma_{4_{[r]}}T_{H[r]} - bT_{H[r]} - a_4T_{H[r]}\left(\frac{D_{[w]}}{T_{H[r]}+D_{[w]}}\right)$$

$$\frac{d}{dt}T_{H[a]} = -\Gamma_{4_{[a]}}T_{H[a]} + bT_{H[r]} + a_4(1-d)T_{H[r]}\left(\frac{D_{[w]}}{T_{H[r]}+D_{[w]}}\right) - \sigma T_{H[a]}\left(\frac{V_w+V_{48}}{T_{H[a]}+V_w+V_{48}}\right)$$

$$\frac{d}{dt}T_{H[w]} = -\Gamma_{4_{[i]}}T_{H[w]} + a_4dT_{H[r]}\left(\frac{D_{[w]}}{T_{H[r]}+D_{[w]}}\right) + \sigma T_{H[a]}\left(\frac{V_w}{T_{H[a]}+V_w+V_{48}}\right) - kT_{C[a]}\left(\frac{T_{H[w]}}{T_{C[a]}+T_{H[w]}}\right)$$

$$\frac{d}{dt}T_{H[48]} = -\Gamma_{4_{[i]}}T_{H[48]} + \sigma T_{H[a]}\left(\frac{V_{48}}{T_{H[a]}+V_w+V_{48}}\right)$$

$$\frac{d}{dt}T_{C[r]} = \rho_8\Theta\left(C^0_{8_{[r]}} - T_{C[r]}\right) - \Gamma_{8_{[r]}}T_{C[r]} - a_8T_{C[r]}\left(\frac{T_{H[w]}}{T_{C[r]}+T_{H[w]}}\right) - a_{8D}T_{C[r]}\left(\frac{D_{[w]}}{T_{C[r]}+D_{[w]}}\right) - a_{8'}T_{C[r]}\left(\frac{T_{H[a]}}{T_{C[r]}+T_{H[a]}}\right)$$

$$\frac{d}{dt}T_{C[Pr1]} = -\Gamma_{8_{[pr]}}T_{C[Pr1]} + a_8T_{C[r]}\left(\frac{T_{H[w]}}{T_{C[r]}+T_{H[w]}}\right) + a_{8D}T_{C[r]}\left(\frac{D_{[w]}}{T_{C[r]}+D_{[w]}}\right) - a_{8'}T_{C[Pr1]}\left(\frac{T_{H[a]}}{T_{C[Pr1]}+T_{H[a]}}\right)$$

$$\frac{d}{dt}T_{C[Pr2]} = -\Gamma_{8_{[pr]}}T_{C[Pr2]} - a_8T_{C[Pr2]}\left(\frac{T_{H[w]}}{T_{C[Pr2]}+T_{H[w]}}\right) - a_{8D}T_{C[Pr2]}\left(\frac{D_{[w]}}{T_{C[Pr2]}+D_{[w]}}\right) + a_{8'}T_{C[r]}\left(\frac{T_{H[a]}}{T_{C[r]}+T_{H[a]}}\right)$$

$$\frac{d}{dt}T_{C[a]} = -\Gamma_{8_{[a]}}T_{C[a]} + a_8T_{C[Pr2]}\left(\frac{T_{H[w]}}{T_{C[Pr2]}+T_{H[w]}}\right) + a_{8D}T_{C[Pr2]}\left(\frac{D_{[w]}}{T_{C[Pr2]}+D_{[w]}}\right) + a_{8'}T_{C[Pr1]}\left(\frac{T_{H[a]}}{T_{C[Pr1]}+T_{H[a]}}\right)$$

$$\frac{d}{dt}D_{[r]} = \rho_D\Theta\left(C^0_{D[r]} - D_{[r]} - D_{[w]} - D_{[48]}\right) - \Gamma_{D_{[r]}}D_{[r]} - \sigma_D D_{[r]}\left(\frac{V_w+V_{48}}{D_{[r]}+V_w+V_{48}}\right)$$

$$\frac{d}{dt}D_{[w]} = -\Gamma_{D_{[a]}}D_{[w]} + \sigma_D D_{[r]}\left(\frac{V_w}{D_{[r]}+V_w+V_{48}}\right)$$

$$\frac{d}{dt}D_{[48]} = -\Gamma_{D_{[a]}}D_{[48]} + \sigma_D D_{[r]}\left(\frac{V_{48}}{D_{[r]}+V_w+V_{48}}\right)$$

$$\frac{d}{dt}V_{[w]} = -\Gamma_V V_{[w]} - \sigma T_{H[a]}\left(\frac{V_w}{T_{H[a]}+V_w+V_{48}}\right) - \sigma_D D_{[r]}\left(\frac{V_w}{D_{[r]}+V_w+V_{48}}\right) + (1-\mu)\alpha T_{H[w]}$$

$$\frac{d}{dt}V_{[48]} = -\Gamma_V V_{[48]} - \sigma T_{H[a]}\left(\frac{V_{48}}{T_{H[a]}+V_w+V_{48}}\right) - \sigma_D D_{[r]}\left(\frac{V_{48}}{D_{[r]}+V_w+V_{48}}\right) + \alpha T_{H[48]} + \mu\alpha T_{H[w]}$$

Control model

Description

The control model is identical to the HIV model except for the fact that the virus infects a cell type C which is not part of the cellular immune system, instead of T_H cells. C cells replicate with rate constant ρ_C if their concentration is below their homeostatic goal, i.e. if $C_C^0 - C_{[r]} - C_{[w]} - C_{[48]} > 0$, and do not replicate otherwise. Since C cells do not need to be activated to become susceptible to the virus, the pool of available cells is higher than in the HIV model. To compensate for this, we adjusted the homeostatic goal for C cells to obtain the same number of susceptible cells in the HIV and control models at set point, and thus obtain similar set-point viral titers.

Equations

$$\frac{d}{dt}T_{H[r]} = \rho_4\Theta\left(C_{4_{[r]}}^0 - T_{H[r]}\right) - \Gamma_{4_{[r]}}T_{H[r]} - bT_{H[r]} - a_4 T_{H[r]}\left(\frac{D_{[w]}}{T_{H[r]}+D_{[w]}}\right)$$

$$\frac{d}{dt}T_{H[a]} = -\Gamma_{4_{[a]}}T_{H[a]} + bT_{H[r]} + a_4 T_{H[r]}\left(\frac{D_{[w]}}{T_{H[r]}+D_{[w]}}\right)$$

$$\frac{d}{dt}T_{C[r]} = \rho_8\Theta\left(C_{8_{[r]}}^0 - T_{C[r]}\right) - \Gamma_{8_{[r]}}T_{C[r]} - a_8 T_{C[r]}\left(\frac{C_{[w]}}{T_{C[r]}+C_{[w]}}\right) - a_{8D}T_{C[r]}\left(\frac{D_{[w]}}{T_{C[r]}+D_{[w]}}\right) - a_{8'}T_{C[r]}\left(\frac{T_{H[a]}}{T_{C[r]}+T_{H[a]}}\right)$$

$$\frac{d}{dt}T_{C[Pr1]} = -\Gamma_{8_{[pr]}}T_{C[Pr1]} + a_8 T_{C[r]}\left(\frac{C_{[w]}}{T_{C[r]}+C_{[w]}}\right) + a_{8D}T_{C[r]}\left(\frac{D_{[w]}}{T_{C[r]}+D_{[w]}}\right) - a_{8'}T_{C[Pr1]}\left(\frac{T_{H[a]}}{T_{C[Pr1]}+T_{H[a]}}\right)$$

$$\frac{d}{dt}T_{C[Pr2]} = -\Gamma_{8_{[pr]}}T_{C[Pr2]} - a_8 T_{C[Pr2]}\left(\frac{C_{[w]}}{T_{C[Pr2]}+C_{[w]}}\right) - a_{8D}T_{C[Pr2]}\left(\frac{D_{[w]}}{T_{C[Pr2]}+D_{[w]}}\right) + a_{8'}T_{C[r]}\left(\frac{T_{H[a]}}{T_{C[r]}+T_{H[a]}}\right)$$

$$\frac{d}{dt}T_{C[a]} = -\Gamma_{8_{[a]}}T_{C[a]} + a_8 T_{C[Pr2]}\left(\frac{C_{[w]}}{T_{C[Pr2]}+C_{[w]}}\right) + a_{8D}T_{C[Pr2]}\left(\frac{D_{[w]}}{T_{C[Pr2]}+D_{[w]}}\right) + a_{8'}T_{C[Pr1]}\left(\frac{T_{H[a]}}{T_{C[Pr1]}+T_{H[a]}}\right)$$

$$\frac{d}{dt}D_{[r]} = \rho_D\Theta\left(C_{D[r]}^0 - D_{[r]} - D_{[48]}\right) - \Gamma_{D_{[r]}}D_{[r]} - \sigma_D D_{[r]}\left(\frac{V_w+V_{48}}{D_{[r]}+V_w+V_{48}}\right)$$

$$\frac{d}{dt}D_{[w]} = -\Gamma_{D_{[a]}}D_{[w]} + \sigma_D D_{[r]}\left(\frac{V_w}{D_{[r]}+V_w+V_{48}}\right)$$

$$\frac{d}{dt}D_{[48]} = -\Gamma_{D_{[a]}}D_{[48]} + \sigma_D D_{[r]}\left(\frac{V_{48}}{D_{[r]}+V_w+V_{48}}\right)$$

$$\frac{d}{dt}V_{[w]} = -\Gamma_V V_{[w]} - \sigma C\left(\frac{V_w}{C+V_w+V_{48}}\right) - \sigma_D D_{[r]}\left(\frac{V_w}{D_{[r]}+V_w+V_{48}}\right) + (1-\mu)\alpha C_{[w]}$$

$$\frac{d}{dt}V_{[48]} = -\Gamma_V V_{[48]} - \sigma C\left(\frac{V_{48}}{C+V_w+V_{48}}\right) - \sigma_D D_{[r]}\left(\frac{V_{48}}{D_{[r]}+V_w+V_{48}}\right) + \alpha C_{[48]} + \mu\alpha C_{[w]}$$

$$\frac{d}{dt}C = \rho_C\Theta\left(C_C^0 - C_{[r]} - C_{[w]} - C_{[48]}\right) - \Gamma_C C - \sigma C\left(\frac{V_w+V_{48}}{C+V_w+V_{48}}\right)$$

$$\frac{d}{dt}C_{[w]} = -\Gamma_{C_{[i]}}C_{[w]} + \sigma C\left(\frac{V_w}{C+V_w+V_{48}}\right) - kT_{C[a]}\left(\frac{C_{[w]}}{T_{C[a]}+C_{[w]}}\right)$$

$$\frac{d}{dt}C_{[48]} = -\Gamma_{C_{[i]}}C_{[48]} + \sigma C\left(\frac{V_{48}}{C+V_w+V_{48}}\right)$$

References

1. Macallan DC, Asquith B, Irvine AJ, Wallace DL, Worth A, et al. (2003) Measurement and modeling of human T cell kinetics. Eur J Immunol 33: 2316-2326.
2. Bofill M, Janossy G, Lee CA, MacDonald-Burns D, Phillips AN, et al. (1992) Laboratory control values for CD4 and CD8 T lymphocytes. Implications for HIV-1 diagnosis. Clin Exp Immunol 88: 243-252.
3. Ho DD, Neumann AU, Perelson AS, Chen W, Leonard JM, et al. (1995) Rapid turnover of plasma virions and CD4 lymphocytes in HIV-1 infection. Nature 373: 123-126.
4. Markowitz M, Louie M, Hurley A, Sun E, Di MM, et al. (2003) A novel antiviral intervention results in more accurate assessment of human immunodeficiency virus type 1 replication dynamics and T-cell decay in vivo. J Virol 77: 5037-5038.
5. Perelson AS, Neumann AU, Markowitz M, Leonard JM, Ho DD (1996) HIV-1 dynamics in vivo: virion clearance rate, infected cell life-span, and viral generation time. Science 271: 1582-1586.
6. Rodrigo AG, Shpaer EG, Delwart EL, Iversen AK, Gallo MV, et al. (1999) Coalescent estimates of HIV-1 generation time in vivo. Proc Natl Acad Sci USA 96: 2187-2191.
7. Wei X, Ghosh SK, Taylor ME, Johnson VA, Emini EA, et al. (1995) Viral dynamics in human immunodeficiency virus type 1 infection. Nature 373: 117-122.
8. Althaus CL, De Vos AS, de Boer RJ (2009) Reassessing the human immunodeficiency virus type 1 life cycle through age-structured modeling: life span of infected cells, viral generation time, and basic reproductive number, R0. J Virol 83: 7659-7667.
9. Haller HJ, Zapas JL, Elias EG (2005) Dendritic cell counts in the peripheral blood of healthy adults. Am J Hematol 78: 314-315.
10. Goonetilleke N, Liu MK, Salazar-Gonzalez JF, Ferrari G, Giorgi E, et al. (2009) The first T cell response to transmitted/founder virus contributes to the control of acute viremia in HIV-1 infection. J Exp Med 206: 1253-1272.
11. Chen HY, Di MM, Perelson AS, Ho DD, Zhang L (2007) Determination of virus burst size in vivo using a single-cycle SIV in rhesus macaques. Proc Natl Acad Sci USA 104: 19079-19084.

12. De Boer RJ, Ribeiro RM, Perelson AS (2010) Current estimates for HIV-1 production imply rapid viral clearance in lymphoid tissues. PLoS Comput Biol 6: e1000906.
13. Hockett RD, Kilby JM, Derdeyn CA, Saag MS, Sillers M, et al. (1999) Constant mean viral copy number per infected cell in tissues regardless of high, low, or undetectable plasma HIV RNA. J Exp Med 189: 1545-1554.
14. Reilly C, Wietgrefe S, Sedgewick G, Haase A (2007) Determination of simian immunodeficiency virus production by infected activated and resting cells. AIDS 21: 163-168.
15. Ramratnam B, Bonhoeffer S, Binley J, Hurley A, Zhang L, et al. (1999) Rapid production and clearance of HIV-1 and hepatitis C virus assessed by large volume plasma apheresis. Lancet 354: 1782-1785.
16. Sanjuán R, Nebot MR, Chirico N, Mansky LM, Belshaw R (2010) Viral mutation rates. J Virol 84: 9733-9748.
